# Neonatal screening for congenital hypothyroidism in an Italian Centre: a 5-years real-life retrospective study

**DOI:** 10.1186/s13052-021-01053-0

**Published:** 2021-05-05

**Authors:** Maria Cristina Maggio, Saveria Sabrina Ragusa, Tommaso Silvano Aronica, Orazia Maria Granata, Eleonora Gucciardino, Giovanni Corsello

**Affiliations:** 1 Department of Health Promotion Sciences, Maternal and Infantile Care, Internal Medicine and Medical Specialities PROMISE, “G. D’Alessandro”, University of Palermo, Via del Vespro 133, 90127 Palermo, Italy; 2 U.O.S. Screening Neonatale e Metabolico Allargato of the Children Hospital “G. Di Cristina”, ARNAS, Palermo, Italy

**Keywords:** Congenital hypothyroidism, Neonatal screening, Twins, TSH, Iodine deficiency

## Abstract

**Introduction:**

Congenital hypothyroidism is an endocrine disease with a significant incidence in the general population (1:2000–1:3000 newborns in Italy) and a different geographical distribution, partially explained by endemic iodine deficiency, genetic traits and autoimmune thyroid diseases.

**Objectives:**

Aims of this study are: to evaluate the incidence of positive neonatal blood spot screening for CH in western Sicily, identified by the screening centre of the Children Hospital “G. Di Cristina”, ARNAS, Palermo; to evaluate the impact of a lower TSH cutoff in the neonatal blood spot screening for CH.

**Materials and methods:**

The TSH threshold of the neonatal screening was established as ≥6 mU/L of whole blood.

We analysed the screening centre data in the period January 2013–April 2018, for a total number of 85.373 babies (45.7% males; 54.3% females).

**Results:**

4.082 Babies (4.8%) required a second screening. Among these, 372 (0.44%) were out of range. The diagnosis of congenital hypothyroidism (CH) was confirmed in 182 babies (0.21%). 77/372 newborns (20.7%) with confirmed high TSH levels showed whole blood TSH levels ≥6 - < 7 mU/L.

In synthesis, 48.9% of the out of range re-testing had a confirmed diagnosis of CH.

**Conclusion:**

The reduction of TSH cutoff to 6 mU/L allowed to identify 77/372 neonates (20.7%) with confirmed out of range TSH, otherwise not recruited by the previously employed TSH cutoff.

## Introduction

Congenital hypothyroidism (CH) occurs in approximately 1:2000–1:3000 neonates in Italy, with possible geographical differences, attributable to the genetic background, to a different incidence of autoimmune thyroiditis between young adult women and to the presence of endemic zones in the region characterized by iodine deficiency.

CH may be permanent or transient: this condition needs a well-defined follow up, concorded with the family. An appropriate follow up allows to decide to try discontinuing l-thyroxine treatment, especially in children with mild increase of TSH levels in neonatal age, in preterm infants, in sons of mothers with autoimmune thyroiditis.

Children with CH will develop severe intellectual disability, showing an intelligence quotient lower than 70. However, more than 95% of neonates with CH have few, if any, clinical manifestations of hypothyroidism. For this reason, neonatal screening allows the precocious diagnosis of CH and supports timely the l-thyroxine replacement therapy preventing or mitigating this intellectual disability [1].

The prognosis of infants identified by screening and who started the treatment early is excellent, with IQ similar to controls. A lower neurocognitive outcome may occur in those infants who started l-thyroxine later (> 30 days of age), or with lower l-thyroxine doses than currently recommended, and in those infants with more severe CH.

Lowering the TSH cutoff was the most important factor contributing to increasing the number of diagnoses of CH in Italy. Irreversible effects of CH on central nervous system development can be reversible in case of a treatment without delay; hence, a neonatal mass screening program provides the best instrument for early diagnosis and treatment [1].

Neonatal screening should detect all forms of primary CH: mild, moderate, and severe. However, those patients with severe CH in whom morbidity is high need a prompt diagnosis permitted only by neonatal screening. The most sensitive test for the identification of primary CH is the determination of TSH blood levels. The best “window” for testing is between 48 and 72 h of age. Blood is spotted onto blood collection paper, dried and eluted into a buffer for TSH analysis. This method identifies primary CH more successfully than primary T4 screening.

The goal of an efficient neonatal blood spot screening program is to perform an early diagnosis and treatment in children with CH: this strategy successfully prevents delayed growth and neurological deficiency [1–3].

Aims of this study are to assess:

- the prevalence of CH in western Sicily, evaluated by the single screening centre of the Children Hospital “G. Di Cristina”, ARNAS, Palermo, Italy;
- the impact of a lower cutoff for TSH in the identification of neonates with CH and the estimation of potential missing diagnosis with a higher cutoff [4, 5].

## Materials and methods

All infants with screening blood spot TSH (capillary whole blood) ≥ 6 mU/L underwent confirmatory diagnostic testing.

We performed a retrospective analysis of neonatal screening data, diagnosis and treatment procedures performed for CH and collected in one of the two Sicily’s screening centers from January 2013 to April 2018. All the neonates who showed the first screening out of range, were retested. In consideration of TSH values and the age of the patient, the retesting was performed at the same time of the TSH, fT3, fT4, anti-thyroglobulin antibodies, anti-thyroid peroxidase antibodies serum dosage. When the diagnosis of CH was confirmed, the patients underwent thyroid scan and/or thyroid scan and scintigraphy.

The neonates with a confirmed diagnosis of CH started treatment with L-thyroxine when the diagnosis was confirmed and are still followed in our paediatric endocrinology unit.

We considered the possible TSH cutoff and compared the number of CH diagnosis if the cutoff was considered as ≥6 - < 7 mU/L; ≥7 - < 10 mU/L; ≥10 mU/L and compared the number and the percentage of neonates resulted positive to the screening every year. We considered with the three possible cutoffs the number of confirmed permanent or transient CH who started treatment, as well.

## Results

A total number of 85.373 babies (45.7% males; 54.3% females) were screened. 11.3% were preterm neonates. 12.2% were sons of immigrant mothers, highlighting the high incidence of multiethnicity of the population. 3% had malformations in association with CH. 4.082 neonates (4.8%) were recalled for a second screening, in consideration of out of range or uncertain results (borderline values, comorbidities, preterm birth, etc). Among these, 372 (0.44%) were out of range. The diagnosis of congenital hypothyroidism (CH) was confirmed in 182 babies (0.21%). In synthesis, 48.9% of the out of range re-testing had a confirmed diagnosis of CH.

The confirmed diagnosis of CH in the period of the study was catalogued considering the birth year, as shown in Table 1.

**Table 1:** Cases of CH in the different years, according to TSH value

Year	Screening number	Second screening/screening number	Increased TSH/second screening	TSH ≥6- < 7 mU/L /increased TSH	TSH ≥7- < 10 mU/L /increased TSH	TSH ≥10 mU/L /increased TSH
2013	17.472	941 (5.4%)	50 (5.3%)	7 (14%)	17 (34%)	26 (52%)
2014	16.020	680 (4.2%)	40 (5.8%)	3 (7.5%)	13 (32.5%)	24 (60%)
2015	15.502	627 (4%)	62 (48.8%)	10 (16.1%)	20 (32.2%)	32 (51.6%)
2016	15.670	659 (4.2%)	80 (12.1%)	19 (23.7%)	26 (32.5%)	35 (43.7%)
2017	15.037	838 (5.6%)	98 (11.6%)	28 (28.5%)	40 (40.8%)	30 (30.6%)
2018 (Jan-Apr)	5.672	337 (5.9%)	42 (12.5%)	10 (23.8%)	17 (40.4%)	15 (35.7%)

The incidence of agenesis varied from a peak of 16% in 2014 to a nadir of 0% thyroid aplasia in 2017–2018 (see Table 2). However, the numbers of thyroid agenesis are too low to settle any conclusion.

**Table 2:** Different distribution per year of agenesis, hypoplasia or thyroid in situ in patients with confirmed diagnosis of CH

Year	Confirmed diagnosis of CH/high TSH at the second screening	agenesis	hypoplasia	Ectopic gland	Thyroid in situ
2013	32 (64%)	3 (9.4%)	7 (21.87%)	1 (3.1%)	21 (65.6%)
2014	25 (62.5%)	4 (16%)	9 (36%)	0	12 (48%)
2015	37 (59.6%)	2 (5.4%)	8 (21.6%)	0	27 (72.9%)
2016	42 (52.5%)	2 (4.8%)	3 (7.1%)	1 (2.4%)	36 (85.7%)
2017	32 (32.6%)	0	5 (15.6%)	1 (3.1%)	26 (81.2%)
2018 (Jan-Apr)	14 (33.3%)	0	2 (14.3%)	1 (7.1%)	11 (78.5%)

The 6% of patients with a confirmed diagnosis of CH were preterm neonates: among those, gestational age ranged between 26 and 34 weeks and birth weight ranged between 800 and 1500 g; 4.9% were twins [6] with a concordance of the diagnosis of CH of 44.4%. Furthermore, anamnestic records revealed that 38.2% were born by a caesarean delivery. 12% had the mother with an autoimmune thyroid disease; 29% had one or more first and/or second-degree relatives affected by thyroid dysfunction.

Twins with a diagnosis of CH, followed in our centre, are all still treated with L-thyroxine.

In 3.8% of all the patients with confirmed hypothyroidism, other congenital anomalies were documented, mainly cardiac malformations. Three childrens showed cardiac defects, 2 patients showed genitourinary system malformations, 1 patient was born with esophageal atresia. Nevertheless, these patients had no documented genetic or chromosomal syndromes.

In order to evaluate the influence of the TSH value cutoff at the screening, we split the sample according to TSH spot levels per volume of whole blood, as performed in neonatal screening (≥6 - < 7 mU/L; ≥7 - < 10 mU/L; ≥10 mU/L). In the 3 groups we evaluated the total number of babies with confirmed CH, transient or permanent (Table 1).

The diagnosis of thyroid in situ, aplastic thyroid or hypoplastic thyroid varied in the different years, however with a significant prevalence of thyroid in situ (Table 2).

Serum TSH levels per serum equivalent volume were the highest in neonates with thyroid agenesis; further, in the group of patients with thyroid hypoplasia, TSH serum levels were higher than in neonates with thyroid in situ (Table 3).

**Table 3:** Bloodspot and serum TSH levels in correlation with thyroid morphology

	Thyroid agenesis (Median: range)	Thyroid hypoplasia (Median: range)	Thyroid in situ (Median: range)
Bloodspot TSH levels (mU/L) (first screening)	134: 6.2–319	16.8: 4.2–266.7	9.4: 0.85–225
Bloodspot TSH levels (mU/L) (second screening)	157: 9.3–462	17.9: 9.3–231	14.5: 21.39–241
Serum TSH levels (μIU/ml)	354: 98.8–940	63.1: 9.4–980	29.8: 0.7–13.2

The neonates with a confirmed diagnosis of CH started treatment with L-thyroxine before the 14th day of life (45.3%), before the 21st day of life (41.2%), after the 21st day of life (13.5%) when the diagnosis was confirmed later (preterm infants, late increase of TSH, etc), and are still followed in our paediatric endocrinology unit. At the date, 73.9% are still in treatment with L-thyroxine.

170/182 among the patients who started L-thyroxine are still followed in our operative unit. The mean TSH value, 14 days after L-thyroxine treatment was started, was 3.38 μU/ml (range: 0.02–14.03), with the prompt and progressive normalization of endocrine profile.

11% of the neonates who started treatment with L-thyroxine had the mother with an autoimmune thyroid disease; otherwise, the 30% had one or more between the first and/or the second-degree relatives affected by thyroid dysfunction.

The reduction of TSH cutoff to 6 mU/L allowed to identify 77/372 neonates (20.7%) with confirmed out of range TSH. These patients would have been missed at the screening if the cutoff had been higher.

Some patients were found out, even if they showed a TSH level lower than 6 mU/L at the first screening (Table 3), because their screening was performed again some days later and the serum TSH levels were detected. These patients were: neonates who underwent a too early discharge from the hospital; preterm or neonates at risk to develop hypothyroidism for autoimmune thyroiditis of the mother; twins with pathological neonatal screening or a confirmed diagnosis of CH.

## Discussion

Considering the high rate of CH in our population of western Sicily, we stratified the sample analysed according to TSH levels relieved at the screening. We confirmed that a significant number of babies could not be detected by the screening if a cutoff > 7 mU/L is chosen. In fact, 20.7% of the patients, otherwise confirmed as CH, showed TSH blood spot levels ≥6 - < 7 mU/L.

Recall rates in different centres for CH neonatal screening range from 0.01 to 13.3%; the difference is linked to various screening methods as screening protocols (use of TSH and T4 or TSH alone), different laboratory techniques and kits, site of sample collection, and different cutoffs. Iodine status is a relevant factor: the control of iodine deficiency, and the use of iodine free antiseptic during delivery are contributing factors for the raise of TSH levels. Recall rate is higher in counties with a higher CH incidence [7] and both are documented in our population.

Iodine deficiency is the most important cause of CH worldwide. Iodine is essential for thyroid hormone synthesis and is present in air, soil and water.

The high incidence of positive anamnestic records confirming thyroid disease in the mother and/or in first- and/or second-degree relatives, supports the hypothesis that the high incidence of CH in Sicily could be linked to geographical and/or endemic conditions, genetic background or lack of iodine supplementation in rural or urban areas where iodine deficiency is still endemic, because of the low iodine concentrations in water and other iodine sources. In fact, western Sicily has several small towns historically built on hills or mountains, with iodine deficiency. The Iodine supplementation campaign was well done in the past decades, however residents reduced adherence in the last years [8]. Furthermore, consanguineous unions are more frequent in small towns, especially in rural zones. In this contest, inherited diseases transmission is higher, as CH secondary to enzymatic defects or receptor mutations. Moreover, autoimmune thyroiditis shows a higher incidence in some areas of western Sicily, increasing the risk of transient or permanent CH.

## Conclusions

Transient hypothyroidism may occur frequently, however infants should be treated with L-thyroxine for the first 3 years of life, considering the risk of growth delay and mental retardation, considering abating the danger for neonatal central nervous system by stabilizing thyroid function. A new evaluation after the age of 3 years is required in these patients, after a withdrawal of the replacement therapy with L-thyroxine, to confirm the persistence of CH.

The goal of initial therapy in CH is to minimize neonatal central nervous system exposure to hypothyroidism by normalizing thyroid function, as soon as possible.

Lowering TSH cutoff to 6 mU/L allowed recognising 20.7% of neonates with confirmed high TSH levels, otherwise not recruited by the previously employed TSH cutoff.

With this cutoff, we detected additional cases of permanent CH, a number of which showed defects of thyroid embryogenesis and severe hypothyroidism at the confirmation of the diagnosis by serum levels of fT4, fT3 and TSH and by ultrasound and/or scintigraphy.

However, the age of 3 years was not reached by all the children at the time of the collection of the study data. Considering this, by 3-year follow-up, we have not yet the data to differentiate the number of children with confirmed permanent CH, or the number of children who tried to stop l-thyroxine and the number of children who did not further require L-thyroxine.

Further studies have to confirm the real prevalence of transient hypothyroidism in children positive to the neonatal screening, confirmed by serum levels of TSH, fT4 and fT3 in western Sicily, and the possible prevention strategies necessary to reduce these numbers and ameliorate the follow-up and the clinical outcome of these children.

It is suitable to implement valid interventions to reduce CH, controlling iodine deficiency and employing iodine-free antiseptic solutions during delivery. Furthermore, preterm and/or low birth weight neonates, twins, sons of mothers with autoimmune thyroiditis or diabetes, can show a late increase of TSH levels: in these patients neonatal screening must be repeated at 14 days from birth [9, 10]. Above all, the incidence of CH is higher in multiple deliveries compared with single deliveries and twins frequently show concordance in developing hypothyroidism, when one twin is affected [6]. In these cases, follow-up is needed and must be considered a second thyroid screening and/or serum TSH and fT4 detection at 14 days from birth to avoid delay in the diagnosis. The misdiagnosis potential risk must be clearly explained to the parents and the paediatric endocrinologist must be sure that blood controls are well done also to initially euthyroid twins.

The goal of a CH screening program is to provide the optimal quality of life and the best neurocognitive development for babies with mild as well as with severe CH [2]. The authors hallmark the require of establishing the optimal TSH cutoff in the screening program in various settings, and of achieving screening benefits considering the geographical area peculiarities.

## Data Availability

The datasets used and/or analysed during the current study are available from the corresponding author on reasonable request.

## Abbreviation

CH: Congenital hypothyroidism
